# Association of p21 3′ UTR gene polymorphism with cancer risk: Evidence from a meta-analysis

**DOI:** 10.1038/srep13189

**Published:** 2015-08-17

**Authors:** Jie Li, Zhenzhen Li, Quancheng Kan, Suke Sun, Yidong Li, Suyun Wang

**Affiliations:** 1 Department of Hepatobiliary and Pancreatic Surgery, The First Affiliated Hospital of Zhengzhou University, Henan Province, China; 2 The Institute of Clinical Medicine, The First Affiliated Hospital of Zhengzhou University, Henan Province, China; 3 Department of Pharmacy, The First Affiliated Hospital of Zhengzhou University, Henan Province, China

## Abstract

Numerous studies have investigated the risk of cancer associated with the polymorphism of p21 3′ UTR (rs1059234 C > T), but results have been inconsistent. We performed this meta-analysis to drive a more precise estimation of the association between this polymorphism and risk of cancer. A comprehensive search was conducted to identify all case-control studies of the rs1059234 C > T polymorphism of p21 3′ UTR and cancer susceptibility. A total of eleven eligible studies, including 3,099 cases and 4,354 controls, relating to the rs1059234 polymorphism of p21 3′ UTR to the risk of cancer were identified. Multivariate and univariate methods revealed no association between this polymorphism and cancer risk. However, subgroup analysis by cancer type suggested that rs1059234 C > T polymorphism was associated with increased risk of squamous cell carcinoma of the head and neck (SCCHN) (dominant model CT + TT vs. CC: OR = 1.51, 95% CI = 1.17–1.94). No significant association was found in other subgroup analyses. This meta-analysis suggested that rs1059234 polymorphism of p21 3′ UTR may be associated with increased SCCHN risk. And larger scale primary studies are required to further evaluate the interaction of p21 3′ UTR rs1059234 polymorphism and cancer risk in specific populations.

Cancer is a major health problem in the most parts of the world. Approximately 12.7 million cancer cases and 7.6 million cancer deaths are estimated to occur each year worldwide^1^. The prevention and treatment for cancers caused increasing financial burdens around the world^2^. As a complex disease, cancer is strongly influenced by environmental and genetic factors, of which gene polymorphism is a critical cause for the difference of individual genetic susceptibility to cancer^3^. Identification of the key gene polymorphisms that are associated with cancer risk is essential for predicting individual at risk.

The gene for p21 (CDKN1A) is localized on chromosome 6p21.2. It consists of three exons and two introns and encodes a 21-kDa protein. The translation region lies mainly in exon 2^4^. p21 is the main protein that is required after p53 activation in response to DNA damage. It plays a crucial role in cell cycle control by inhibiting activities of cyclin E_CDK2 and cyclin A_CDK2 complexes. As a result, it leads to dephosphorylation of the RB protein (pRb), which induces G1 arrest conducting to DNA repair or apoptosis^4^,^5^. Due to its vital function in cellular growth, it has been considered that p21 may have an influence in carcinogenesis^6^.

Several studies have shown that p21 polymorphisms may affect protein expression and activity and play a role in susceptibility to cancer^7^,^8^. Two major p21 polymorphisms in codon 31 (p21 C98A, dbSNP rs1801270) and in the 3′ UTR (p21 C70T, dbSNP rs1059234), both alone and/or in combination, may have an effect on carcinogenesis^7^. The p21C98A polymorphism results in non-synonymous serine to arginine substitution in the protein, which affects the DNA-binding zinc finger motif. The other polymorphism, p21 C70T (rs1059234), occurs 20 nucleotides downstream of the stop codon in the 3′ UTR region. This region is considered to be an important site for cell differentiation, proliferation and tumor suppression^9^,^10^,^11^. Hence it affects mRNA stability by inducing rapid message degradation^12^,^13^,^14^,^15^, leading to an alteration in protein expression level^7^,^16^,^17^,^18^.

To date, a number of molecular epidemiological studies have been done to evaluate the association between p21 3′ UTR rs1059234 polymorphism and different types of cancer risk in diverse populations^7^,^19^,^20^,^21^,^22^,^23^,^24^,^25^,^26^,^27^,^28^. However, the results were inconsistent or even contradictory, partially because of the possible small effect of the polymorphism on cancer risk and the relatively small sample size in each of published study. Therefore, we performed a comprehensive meta-analysis to derive a more precise estimation of the relationship between p21 3′ UTR rs1059234 polymorphism and the risk of cancer.

## Results

### Study characteristics

**Figure 1:**
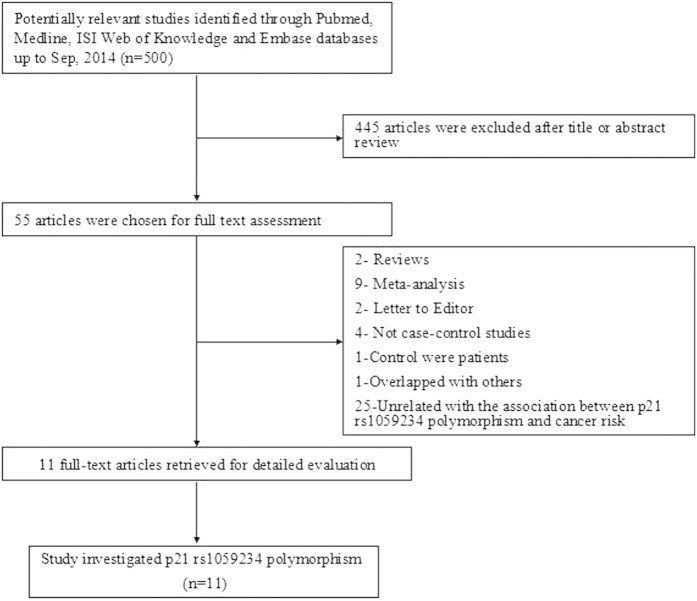
Flow diagram of selection of studies included in the current meta-analysis for the association between p21 3′ UTR rs1059234 polymorphism and cancer risk.

As shown in Fig. 1, a total of 500 relevant articles were retrieved from Pubmed, Medline, ISI Web of Knowledge and Embase databases using search terms described in the methods section. After title and abstract screening, 445 publications, which did not investigate the association between cancer risk and the polymorphism of interest, were excluded; and then, the remaining 55 publications were carefully reviewed according to the criteria described in the ‘materials and methods’ section. Forty-four publications were further removed, among which 25 publications were unrelated with p21 3′ UTR rs1059234 polymorphism and cancer risk, 9 were meta-analysis, 4 were not case-control studies, 2 were reviews, 2 were letter to editor, 1 used cancer patients as controls and last one was overlapped with others. After the removal of all studies that didn’t meet our criteria, a total of 11 eligible studies were finally included in our meta-analysis^7^,^19^,^20^,^21^,^22^,^23^,^24^,^25^,^26^,^27^,^28^. Table 1 listed the main characteristics of these studies. In total, 3099 cases and 4354 controls were included in the pooled analyses. Among these studies, there were 2 colorectal cancer studies, 2 head and neck cancer studies, 2 prostate cancer studies, and 5 studies with the “other cancers” (esophageal cancer, retinoblastoma, endometrial cancer, cervical cancer and ovarian cancer). Of the 11 studies, there were 7 with Caucasians and 4 with Asian ethnicity populations, respectively. The distribution of genotypes in the controls of all studies was consistent with Hardy-Weinberg equilibrium except for two studies^21^,^26^, which was tested in the sensitivity analyses. Furthermore, 5 studies were considered as low quality (quality score ≤9), and 6 were considered as high quality (quality score >9). All of the cases were histological confirmed, and most controls were matched by sex, age and ethnicity.

### Quantitative analysis

**Figure 2:**
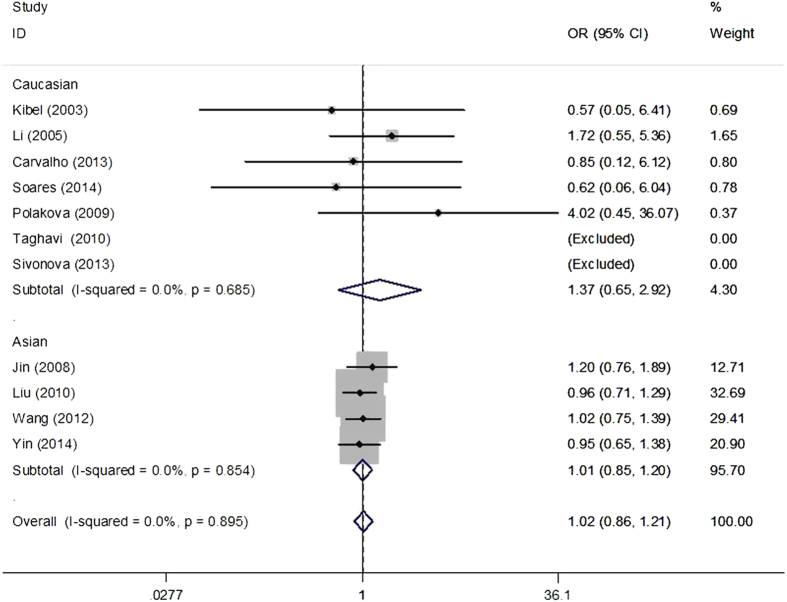
Forest plot of effect estimates for p21 3′ UTR rs1059234 polymorphism and cancer risk in recessive model (TT vs. TC + CC). Each study is shown by the point estimate of the odds ratio, and a horizontal line denotes the 95% confidence interval. The pooled odds ratio is represented by a diamond. The area of the gray squares reflects the weight of the study in the meta-analysis.

**Figure 3:**
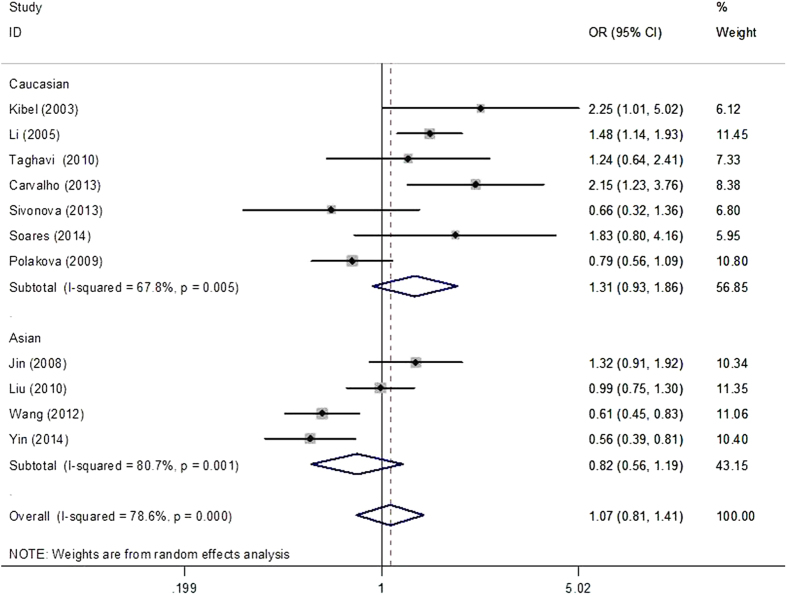
Forest plot of effect estimates for p21 3′ UTR rs1059234 polymorphism and cancer risk in dominant model (TT + TC vs. CC). Each study is shown by the point estimate of the odds ratio, and a horizontal line denotes the 95% confidence interval. The pooled odds ratio is represented by a diamond. The area of the gray squares reflects the weight of the study in the meta-analysis.

The main results of meta-analysis of p21 3′ UTR rs1059234 polymorphism and cancer risk were presented in Table 2. There was no evidence of significant association between p21 3′ UTR rs1059234 polymorphism and cancer risk when all the eligible studies were pooled into the meta-analysis (recessive model: OR = 1.02, 95% CI = 0.86–1.21; dominant model: OR = 1.07, 95% CI = 0.81–1.41, Figs 2 and 3). Both Cochran’s Q test and the estimate of I^2^ revealed significant heterogeneity among the constituent studies. To avoid the influence of heterogeneity among studies, we carried out subgroup analyses for ethnic group, each type of cancer, source of controls and score. We failed to detect significant association between the polymorphisms and cancer risk neither in Caucasian nor in Asian populations (Figs 2 and 3). Interestingly, when stratified by cancer type, marginally increased risks were observed in squamous cell carcinoma of the head and neck (SCCHN) (dominant model: OR = 1.51, 95% CI = 1.17–1.94). Meta-analysis in subgroups according to source of control and quality score did not yield any significant association (Table 2).

### Heterogeneity and sensitivity analyses

**Figure 4:**
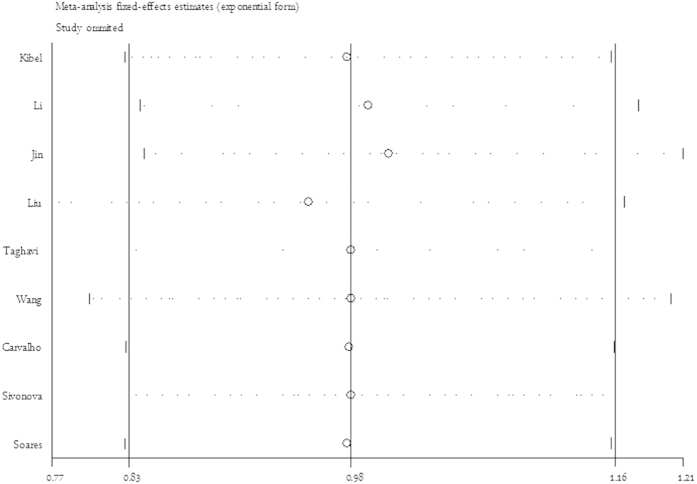
One-way sensitivity analysis of the pooled ORs and 95% CI for p21 3′ UTR rs1059234 in recessive model (TT vs. TC + CC), omitting each dataset in the meta-analysis. Random effect model was used.

As shown in Table 2, substantial heterogeneities were observed among investigations for p21 3′ UTR rs1059234 polymorphism and cancer risk (dominant model: *P*_*he*t_ = 0.000), not the recessive model (*P*_*he*t_ = 0.895). The meta-regression analysis did not yield any significant difference between subgroup analysis (tau^2^ > 0.05). Thus, one-way sensitivity analyses of the pooled odds ratios and 95% confidence intervals for rs1059234 were performed. The pooled ORs were calculated by means of a random effects model. When omitting each dataset in the meta-analysis, the pooled ORs were always persistent. The analyses for carriers of the rs1059234 T-allele versus the CC genotype was shown at Fig. 4. Although the genotype distributions in two studies did not follow Hardy-Weinberg equilibrium, the corresponding pooled OR was not materially altered by excluding the study. Similar, no other single study influenced the pooled ORs qualitatively as indicated by sensitivity analyses, suggesting that the results of this meta-analysis are stable.

### Publication bias

The Begg’s test and Egger’s test were performed to quantitatively evaluate the publication bias of the studies. No significant publication bias was observed in this meta-analysis (TT vs. CC: Begg’s *P* = 1.00, Egger’s *P* = 0.372; CT vs. CC: Begg’s *P* = 0.64, Egger’s *P* = 0.409; dominant model: Begg’s *P* = 0.64, Egger’s *P* = 0.426; recessive model: Begg’s *P* = 0.917, Egger’s *P* = 0.474). The shapes of the funnel plots seemed symmetrical for all analyses (data not shown).

### Multivariate meta-analyses for the association of p21 3′ UTR rs1059234 polymorphism with cancer risk

To validate our results, multivariate meta-analyses were performed. The analysis that was performed revealed no evidence for the association of p21 3′ UTR rs1059234 polymorphism with cancer risk, since the TT vs CC yields a p-value = 0.257 and OR: 0.93 (95% CI: 0.78–1.27) and the CT vs CC, an OR: 0.93 (95% CI: 0.78–1.27) and p-value = 0.628. The multivariate methods produced wider confidence intervals compared to univariate ones. The estimate of λ (0.99, 95% CI = 0.62, 1.45), which was close to 1, suggested that a dominant model was more likely. Nevertheless, these findings were expected since the majority of the univariate tests were unable to show an association. Multivariate meta-analysis could help in avoiding an inflation of the Type I error rate (i.e. Reduce false positive findings), but it does not offer greater statistical power.

## Discussion

Previous studies evaluating the association between p21 3′ UTR rs1059234 polymorphism and cancer risk have provided inconsistent results, and most of these studies involved no more than a few hundred cancer cases, which is too few to assess any genetic effects reliably. Meta-analysis has been recognized as an important way to detect the effect of selected genetic polymorphisms on disease risk precisely and to identify potential important sources of between-study heterogeneity. Hence, we performed this meta-analysis including 11 eligible studies involved 3099 cases and 4354 controls to investigate the association between p21 3′ UTR rs1059234 polymorphism and cancer risk. To the best of our knowledge, this is the first meta-analysis of the association between p21 3′ UTR rs1059234 polymorphism and cancer risk. Our results suggested that the p21 3′ UTR rs1059234 polymorphism (rs1059234, C > T) was not associated with cancer risk when all studies were pooled together. In subgroup analyses stratified by ethnicity, cancer type, source of control, and study quality, statistical significant association was observed just among SCCHN patients.

Cell cycle progression is regulated by cyclin-dependent kinases, crucial for normal growth and differential. Disruption of cell cycle control is common in cancer cells and is believed to play an essential role in cancer initiation and development. The p21 protein binds to and inhibits the activity of cyclin-CDK2 or -CDK4 complexes, and disrupts cell cycle progression at G1 phase^29^,^30^. The expression of p21 is induced by the binding of tumor suppressor protein p53 to the p21 promoter^31^,^32^,^33^. The p21 protein can also interact with proliferating cell nuclear antigen (PCNA), a DNA polymerase accessory factor, and plays a regulatory role in S phase DNA replication and DNA damage repair^34^. The p21 3′ UTR rs1059234 polymorphism is thought to cause a functional change in p21, and as this polymorphism lies in a crucial region for cell differentiation, proliferation may increase cancer risk by altering messenger RNA stability, which, in turn, may affect protein expression and activity^9^,^35^. However, in our study, no significant association between variant genotypes of rs1059234 polymorphism and cancer risk was observed in the pooled analysis, which was inconsistent with the hypothesis above. Multivariate meta-analysis did not change the significance of the results. Several potential concerns should be discussed for the non-significant associations between rs1059234 polymorphism and cancer susceptibility. First, cancer is a multi-factorial disease resulting from complex interactions between environmental and genetic factors^36^. It is possible that the variants at this locus have some modest effects on cancer. Environmental factors, such as living habits and exposure to carcinogens, however, may also play a role in cancer development. Thus, no regard of these factors may confer the non-significance for the independent role of rs1059234 polymorphism in cancer development. Second, the functional role of rs1059234 polymorphism is not yet completely elucidated. Some authors suggest that the 3’ polymorphism may increase the risk of cancer by altering mRNA stability and, in doing so, increase intracellular levels of CDKN1A^21^, and others observed a linkage disequilibrium between rs1059234 and other p21 polymorphisms, namely rs1801270 at codon 31 and rs3176352 in an intron, which have been functionally associated with CDKN1A function and gene expression levels^19^,^37^. This could cover the true associations of p21 gene polymorphisms with cancer. Therefore, other variants as cancer risk factors should be induced as co-variants to determine their true effects. The lack of considering above confounding factors might affect the significance of their results. Moreover, the null result may be due to the limited number of studies included in the meta-analysis, which had insufficient statistical power to detect a slight effect or may have generated a fluctuated risk estimate. Therefore, the negative results of the association between rs1059234 polymorphism and cancer risk should be interpreted with caution.

Furthermore, a significant association was found only in some of the subgroup analyses, such as cancer type. Our meta-analysis does reveal that rs1059234 CT + TT genotypes conferred increased risk to SCCHN but not other cancers. It is suggested that there may be the histological difference in risk, due to certain genotypes conferring a greater susceptibility to a particular histological type of cancer, which can be seen anywhere^38^. In addition, other factors may be modulating the p21 3′ UTR rs1059234 polymorphism functionality. However, the exact mechanism for association between different tumor sites and rs1059234 polymorphism was not clear, carcinogenetic mechanism may also differ by different tumor sites and rs1059234 genetic variants may exert varying effects in different cancers. Our results seem to confirm and establish the trend in the meta-analysis of p21 3′ UTR rs1059234 polymorphism and SCCHN risk that the data by Li *et al.*^7^ had indicated. Our analysis of λ also suggests a tendency towards dominant mode of increased cancer risk effect of p21 3′ UTR rs1059234. However, at any case, the association between rs1059234 polymorphism and SCCHN risk remains an open field, as the number if studies (n = 2) is considerably smaller than that needed for the achievement of robust conclusions.

In the present meta-analysis, between-studies heterogeneity was observed between p21 3′ UTR rs1059234 polymorphism and risk of cancer. Deviation of HWE may reflect methodological problem such as genotyping errors, population stratification or selection bias. When these studies were excluded, the results were not changed among overall cancer and some subgroup analyses, indicating that our meta-analysis was statistically robust. When the meta-analysis was performed excluding studies with small sample sizes and any subgroup analysis, indicating that small sample size did not influence statistically robust.

Our meta-analysis has several strengths. First, a systematic review of the association of p21 3′ UTR rs1059234 polymorphisms with cancer risk is statistically more powerful than any single study. Second, the quality of eligible studies included in current meta-analysis was satisfactory and met our inclusion criterion. Third, we did not detect any publication bias indicating that the whole pooled results should be unbiased. However, although we have put considerable efforts and resources into testing possible association between p21 3′ UTR rs1059234 polymorphisms and cancer risk, there are still some limitations inherited from the published studies. First, our results were based on single-factor estimations without adjustment for other risk factors including environmental factor and other lifestyles. Second, in the subgroup analysis may have had insufficient statistical power to check an association. Third, the controls were not uniformly defined. Some studies used a healthy population as the reference group, whereas others selected hospital patients without organic cancer as the reference group. Therefore, non-differential misclassification bias is possible because these studies may have included the control groups who have different risks of developing cancer of various organs.

In summary, this meta-analysis suggests that no significantly increased cancer risk associated with p21 3′ UTR rs1059234 polymorphism, however, marginally increased risks were observed for rs1059234 in SCCHN. It is necessary to conduct large sample studies using standardized unbiased genotyping methods, homogeneous cancer patients and well-matched controls. Moreover, further studies estimating the effect of gene-gene and gene-environment interactions may eventually lead to our better, comprehensive understanding of the association between p21 3′ UTR rs1059234 polymorphisms and cancer risk.

## Materials and Methods

### Publication search

We searched the literature databases, including Pubmed, Medline, ISI Web of Knowledge and Embase databases. The strategy to identify all eligible articles involved the combined use of the following search items: ‘p21’ or ‘CDKN1A’ or ‘rs1059234’, ‘polymorphism’ or ‘variant’ and ‘cancer’, ‘carcinoma’, ‘tumor’ or ‘neoplasms’ and including all alternative locations and combinations of the terms. Reviews and bibliographies of the relevant studies identified were hand searched to find additional eligible studies, including clinical trials, cohort studies, etc. There was no language and sample limitation, and the literature search was updated on Sep 20, 2014. We evaluated all associated publications to retrieve the most eligible literatures. Their reference lists were hand-searched to find other relevant publications. Of the studies with overlapping data published by the same investigators, only the most recent or complete study was included in this meta-analysis.

### Inclusion and exclusion criteria

The following inclusion criteria were used to select literatures for the meta-analysis: (1) be a case-control study, nested case-control or a cohort study; (2) evaluated the association of p21 3′ UTR rs1059234 genetic polymorphisms with cancer risk, and (3) provide sufficient data for calculating the odds ratio (OR) with 95% confidence interval (95% CI). The major reasons for exclusion of the studies were as follows: (1) not for cancer research, (2) only case population; case reports, conference abstract, reviews, meta-analyses and studies without detailed data, (3) duplicate of previous publication, and (4) control population including malignant tumor patients.

### Data extraction

Two investigators reviewed and extracted information from all eligible publications independently, according to the inclusion and exclusion criteria listed above. An agreement was reached by discussion between the two reviewers whenever there was a conflict. The following items were collected from each study: first author’s surname, year and country of the study, source of control groups (population or hospital-based controls), statistical data, and ethnicity, total number of cases and controls and genotyping method. Different ethnic descents were categorized as Caucasian and Asian.

### Quality assessment

Two investigators assessed the quality of each investigation using the quality assessment (Supplemental Table 1), which was derived from previously published meta-analysis of molecular association studies^39^. Quality scores of studies ranged from 0 (lowest) to 15 (highest). Studies with scores ≤9 were categorized into low quality, while those with scores >9 were considered as high quality. A third investigator (SS) would be involved if there existed any disagreement.

### Statistical analyses

Crude ORs and their corresponding 95% CIs were used to evaluate the strength of associations between p21 3′ UTR rs1059234 polymorphisms and cancer risk. The pooled ORs were estimated for p21 3′ UTR rs1059234 polymorphism under the recessive model (TT vs. CT + CC) and dominant model (TT + CT vs. CC). To evaluate whether results of the data sets were homogeneous, we used Q test^40^. P < 0.05 was considered significant for the heterogeneity. We also calculated the quantity I^2^ that represents the percentage of total variation across studies. As a guide, values of I^2^ less than 25% may be considered “low”, values about 50% may be considered “moderate”, and values more than 75% may be considered “high”^41^. The fixed effects model was used when there was no heterogeneity of the results of studies; otherwise, the random-effects model was used. In the absence of heterogeneity, the two methods provide identical results, because the fixed effects model, using the Mantel-Haenszel’s method, assumes that studies are sampled from populations with the same effect size, making an adjustment to the study weights according to the inter-study variance; whereas the random-effects model using the DerSimonian and Laird’s method assumes that studies are taken from populations with different effect sizes, calculating the study weights both from inter-study and between-study variances, considering the extent of variation or heterogeneity. The departure of frequencies from expected under Hardy Weinberg equilibrium was assessed by X^2^ goodness of fit tests in controls. In addition to the comparison among all subjects, we also performed stratification analyses by cancer type (if one cancer type contained less than two individual studies, it was combined into the “other cancers” group).

The multivariate random-effects method of meta-analysis was also applied as a more advanced method for testing gene-disease associations. In this framework, the two summary log-odds ratios related to the risk allele, e.g. the log-odds ratio of heterozygotes vs. homozygotes (AB vs. AA) and the log-odds ratio of homozygotes for the risk allele vs. homozygotes for the wild type allele (BB vs. AA), are modeled simultaneously as a bivariate response. This method has several important properties, since it can infer and quantify the genetic model of inheritance directly, by estimating the ratio λ of the two log-odds ratios^42^,^43^,^44^. This way, we avoid multiple testing and thus the inflation of the Type I error rate.

Begg and Mazumdar adjusted rank correlation test and Egger’s linear regression test were used to provide diagnosis of the potential publication bias. Sensitivity analyses were also performed to assess the stability of the results, namely, a single study in the meta-analysis was deleted each time to manifest the influence of the individual dataset to the pooled odds radio (OR). Stata Version 12.0 (Stata Corp LP, College Station, TX, USA) was the statistical package that was used for all analyses. Results with *p*-value < 0.05 were considered statistical significant.

## Additional Information

**How to cite this article**: Li, J. *et al.* Association of p21 3' UTR gene polymorphism with cancer risk: Evidence from a meta-analysis. *Sci. Rep.* **5**, 13189; doi: 10.1038/srep13189 (2015).

## Supplementary Material

Supplementary Information

## Figures and Tables

**Table 1:** Main characteristics of all studies included in the meta-analysis.

**First author**	**Year**	**Country**	**Ethnicity**	**Cancer type**	**Genotyping method**	**Source of control**	**Total sample size (case/control)**	**HWE**	**Score**
Kibel	2003	USA	Caucasian	Prostate cancer	PCR-RFLP	HB	96/106	0.007	6
Li	2005	USA	Caucasian	SCCHN	PCR-RFLP	HB	712/1222	0.445	13
Jin	2008	China	Asian	Ovarian cancer	PCR-RFLP	PB	161/284	0.661	11
Liu	2010	China	Asian	Colorectal cancer	PCR-RFLP	PB	373/838	0.16	13
Taghavi	2010	Iran	Caucasian	ESCC	PCR-RFLP	HB	126/100	0.323	9
Wang	2012	China	Asian	Cervical cancer	PCR-RFLP	HB	393/434	0.694	8
Carvalho	2013	Brazil	Caucasian	Retinoblastoma	PCR-RFLP	HB	141/120	0.66	5
Sivonova	2013	Slovak	Caucasian	Prostate cancer	PCR-RFLP	HB	118/130	0.292	10
Soares	2014	Portugal	Caucasian	SCCHN	PCR-RFLP	PB	102/191	0.000	10
Yin	2014	China	Asian	EC	PCR-RFLP	HB	263/315	0.383	8
Polakova	2009	Czech	Caucasian	Colorectal cancer	PCR-RFLP	HB	614/614	0.16	12

Abbreviations: SCCHN, squamous cell carcinoma of the head and neck; ESCC, esophageal squamous cell carcinoma; EC, endometrial cancer; PCR-RFLP, polymerase chain reaction-restriction fragment length polymorphism; PB, population-based; HB, hospital-based; HWE: Hardy-Weinberg equilibrium of controls.

**Table 2:** Univariate meta-analysis results of the association between p21 3′ UTR rs1059234 polymorphism and cancer risk.

			**Dominant TT + CT versus CC**			**Recessive TT versus CT + CC**		
	**N**	**Cases/controls**	**OR (95% CI)**	***P***_***het***_	***P***_***Z***_	**OR (95% CI)**	***P***_***het***_	***P***_***Z***_
Total	11	3099/4354	1.07 (0.81–1.41)^a^	0.000	0.622	1.02 (0.86–1.21)	0.895	0.802
Ethnicity								
Caucasian	7	1909/2483	1.31 (0.93–1.86)^a^	0.005	0.125	1.37 (0.65–2.92)	0.685	0.408
Asian	4	1190/1871	0.82 (0.56–1.19)^a^	0.001	0.290	1.01 (0.85–1.20)	0.854	0.946
Cancer types								
PCa	2	214/236	1.20 (0.36–3.99)^a^	0.026	0.763	0.57 (0.05–6.41)	NA	0.65
SCCHN	2	814/1413	1.51 (1.17–1.94)	0.635	0.001	1.37 (0.51–3.70)	0.43	0.536
CRC	2	987/1452	0.90 (0.73–1.11)	0.293	0.330	0.99 (0.74–1.33)	0.203	0.949
Others types^b^	5	1084/1253	1.00 (0.62–1.64)^a^	0.000	0.986	1.03 (0.83–1.27)	0.885	0.785
Source of controls								
HB	8	2463/3041	1.02 (0.70–1.48)^a^	0.000	0.921	1.03 (0.82–1.29)	0.774	0.826
PB	3	636/1313	1.13 (0.91–1.40)	0.241	0.257	1.02 (0.79–1.30)	0.656	0.896
Score								
Low	5	1019/1075	1.09 (0.62–1.91)^a^	0.000	0.773	0.98 (0.78–1.25)	0.959	0.884
High	6	2080/3279	1.10 (0.85–1.43)^a^	0.019	0.472	1.06 (0.84–1.35)	0.547	0.612

*P*_*het*_, *P* Values for heterogeneity from Q test. *P*_*Z*_, *P* Values for overall effect. The bold values mean that their association is significant; CRC, Colorectal cancer; SCCHN, squamous cell carcinoma of the head and neck; PCa, Prostate cancer; PB, population-based; HB, hospital-based; NA, not applicable; ^a^Random effect model was used when *P*_*het*_ < 0.05; ^b^Other cancers including esophageal cancer, retinoblastoma, endometrial cancer, cervical cancer and ovarian cancer.
