# Supplementary material for: Association of p21 3′ UTR gene polymorphism with cancer risk: Evidence from a meta-analysis
Source: Sci Rep. 2015 Aug 17;5:13189. doi: 10.1038/srep13189 (PMC4538688; doi:10.1038/srep13189)
Supplement: Supplementary Information [file srep13189-s1.doc]

**Association of p21 3′ UTR gene polymorphism with cancer risk: Evidence from a meta-analysis**

Jie Li^1^, Zhenzhen Li^2 *^, Quancheng Kan^2,3^, Suke Sun^2^, Yidong Li^2^, Suyun Wang^2^

1 Department of Hepatobiliary and Pancreatic Surgery, 2 The Institute of Clinical Medicine, 3 Department of Pharmacy, The First Affiliated Hospital of Zhengzhou University, Henan Province, China

*Corresponding author:

Li Zhenzhen, MD., Ph.D

The Institute of Clinical Medicine

The First Affiliated Hospital of Zhengzhou University

Henan, China. 450052.

Tel (Fax): +86 371 66295215.

Email: lzzkiki@126.com

| Supplemental Table 1. Score of quality assessment | |
| --- | --- |
| **Criteria** | **Score** |
| Representativeness of case |  |
| Selected from population cancer registry | 2 |
| Selected from hospital | 1 |
| No method of selection described | 0 |
| Representativeness of control |  |
| Population-based | 3 |
| Blood donors | 2 |
| Hospital-based | 1 |
| Not described | 0 |
| Ascertainment of cancer case |  |
| Histopathologic confirmation | 2 |
| by patient medical record | 1 |
| Not described | 0 |
| Control selection |  |
| Controls matched with cases by age and sex | 2 |
| Controls matched with cases only by age or by sex | 1 |
| Not matched or not descried | 0 |
| Genotyping examination |  |
| Genotyping done blindly and quality control | 2 |
| Only genotyping done blindly or quality control | 1 |
| Unblinded and without quality control | 0 |
| HWE |  |
| HWE in the control group | 1 |
| HWD in the control group or not mentioned | 0 |
| Total sample size |  |
| > 1000 | 3 |
| 501 - 1000 | 2 |
| 201 - 500 | 1 |
| ≤ 200 | 0 |
